# Supplementary material for: Experimental and computer study of the mechanism and identification of conditions for energy-efficient removal of moisture from materials under ultrasonic exposure
Source: PLoS One. 2024 May 31;19(5):e0302585. doi: 10.1371/journal.pone.0302585 (PMC11142529; doi:10.1371/journal.pone.0302585)
Supplement: Attachment — Submitted filename: Response to Reviewers.docx [file pone.0302585.s002.docx]

Many thanks to the reviewer for his valuable comments on the article. My co-authors and I place great value on your comments. We have made detailed changes according to the comments and marked them in green in the revised manuscript.

Below are the reviewer's questions and their answers:

1. The introduction part is divided into too many paragraphs, and the novelty and highlights are not well presented. A proper background knowledge of the area should be presented and what are the shortcomings or gaps in previous research? What inspiration does previous research have for this study?

Answer. Thanks for your recommendations. We have significantly revised the Introduction: we have removed redundant information, focused attention on the problem, and indicated the works that inspired our research.

2. I think the experimental results should be better analyzed by the theoretical study.

Answer. My co-authors and I would like to express our gratitude for giving us the opportunity to finalize the article. We conducted a regression analysis of the experimental data obtained during the drying process. An exponential function was chosen as a mathematical model. The obtained model coefficients and coefficients of determination are presented in Tables 3-5.

3. Ec is the amount of electric energy consumed for the convection drying, however, the electric energy consumed for the ultrasonic drying is denoted by Ec+US, this symbol can easily be misunderstood by readers.

Answer. We are grateful that you drew attention to this annoying shortcoming. We have corrected the designation of electricity spent on ultrasonic drying.

We tried to take into account and correct all your comments. We sincerely hope that our response met your expectations.

Best wishes!
